# Safety assessment of immunization in pregnancy^☆^

**DOI:** 10.1016/j.vaccine.2017.09.033

**Published:** 2017-12-04

**Authors:** Sonali Kochhar, Jorgen Bauwens, Jan Bonhoeffer

**Affiliations:** Global Healthcare Consulting, Delhi, India; Erasmus University Medical Center, Rotterdam, The Netherlands; University of Basel Children’s Hospital, Basel, Switzerland; Brighton Collaboration Foundation, Basel, Switzerland

In pregnancy, immunological and physiological changes may increase a woman’s risk of infections and their sequelae. The immature immune system of the fetus and neonate pose an additional risk of infection and associated complications for the developing infant, including preterm birth [1], [2]. In 2016, it was estimated that neonatal death accounted for approximately 45 percent of mortality among children less than five years of age [3]. Immunization in pregnancy has emerged as an important and successful public health intervention globally to reduce mortality and morbidity among pregnant women, their developing fetuses and neonates, and infants [4]. It may become a key strategy to address neonatal mortality in particular. This is particularly true in low and middle-income countries (LMIC) where the burden of vaccine-preventable diseases is the greatest and access to basic health services is limited.

An important aim of vaccinating pregnant women is to increase pathogen-specific antibodies in the mother to protect against some of the leading causes of morbidity in pregnant women [5]. Also, high and protective levels of immunoglobulin G may be transferred across the placenta from the vaccinated mother to the fetus [6]. This may reduce risk of transmitting infections to the infant and may also directly provide passive immunity early in life, which is a period of vulnerability for the infant [6]. The success of maternal tetanus vaccination demonstrates this principle and is part of routine care in many countries: 41 out of 59 countries achieved Maternal and Neonatal Tetanus (MNT) elimination as a result of the MNT Elimination programme in conjunction with the World Health Organization (WHO) and UNICEF [7]. Influenza and pertussis vaccines are being increasingly recommended as an integral part of immunization in pregnancy programs. These programs have demonstrated the feasibility and effectiveness of immunization in pregnancy programs in high, middle and low-income countries. New vaccines are being developed to prevent infections in pregnant women and infants, including against Group B streptococcus, respiratory syncytial virus, and cytomegalovirus [4].

Immunization in pregnancy is currently an underutilized strategy and public awareness and acceptance could be improved. Despite evidence for their safety and effectiveness in both mothers and their infants, vaccine uptake in pregnancy remains low for influenza and moderate for pertussis vaccine. The uptake of the influenza vaccine in pregnancy rarely exceeds 50 percent in developed countries, even in countries with national vaccination strategies in place. For example, 50 percent of women in the US were vaccinated against influenza just before or during pregnancy in the 2015–16 influenza season [8]. Influenza and pertussis vaccine uptake in pregnancy in England was around 42 percent and 60 percent, respectively in 2015–2016. The UK has among the highest coverage rates globally, indicating the scale of potential improvement [9]. The coverage of seasonal influenza vaccination in the 2014–15 influenza season in pregnant women in five EU Member States was between 0.3% and 56.1% (median 23.6%). No country achieved the EU target of 75 percent coverage among the risk groups) [10]. In LMIC, influenza vaccine coverage amongst pregnant women is negligible in 35 of the 64 tropical countries that recommend seasonal influenza vaccination for pregnant women [11].

Barriers to vaccination in pregnancy are complex and vary depending on the country and population. The safety of vaccines administered during pregnancy is a key consideration for pregnant women, healthcare providers, vaccine manufacturers, investigators, regulators, ethics committees and communities [4]. There is a need for a globally harmonised approach to actively monitor the safety of vaccines used in immunization programs for pregnant women [12].

Historically, there was little standardization of case definitions for adverse events following immunization (AEFI) [13]. This resulted in limited comparisons of safety data across vaccine trials and studies in pre- and post-licensure settings. The Brighton Collaboration (BC) was formed in 2000 to help to overcome this shortcoming [14]. Today, BC case definitions are used and recommended for use by normative bodies such as the World Health Organization (WHO), the US FDA, the European Medicines Agency, the US Centers for Disease Control and Prevention (CDC), and the European Centre for Disease Prevention and Control (ECDC) [15].

The GAIA (Global Alignment of Immunization Safety Assessment in Pregnancy) project (http://gaia-consortium.net), coordinated by the Brighton Collaboration Foundation (BCF) and funded by the Bill and Melinda Gates Foundation, was initiated in 2015 for an initial period of two years (2015–2016). This was a response to the World Health Organization’s call for a globally harmonised approach to actively monitor the safety of vaccines and immunization in pregnancy programs with a specific focus on LMIC needs and requirements [12]. In the GAIA project, experts from 13 organisations (BCF, US National Institute of Health, WHO, Global Healthcare Consulting, University of Washington, Baylor College of Medicine, Monash Institute of Medical Research, St. George’s University of London, Erasmus University Medical Center, Cincinnati Children’s Hospital, Public Health Agency Canada, Synapse Research Management Partners and International Alliance for Biological Standardization) collaborated with over 200 volunteers worldwide who participated in 25 specific working groups [16].

During the GAIA project, a global functional network of experts was created, bringing together experts in vaccinology, maternal health, and neonatology from academia, public health institutes, regulatory agencies, investigators and vaccine manufacturers from LMIC and high-income countries. GAIA outputs include a landscape analysis of available standards and guidance documents, comprising regulatory guidance pertinent to immunization in pregnancy from the Food and Drug Administration (FDA), the European Medicines Agency (EMA) and the International Conference on Harmonisation [4].

Further, GAIA has developed two guideline documents for a harmonised conduct of clinical trials of vaccines in pregnant women [17], [18]. This includes recommendations for harmonised collection, analysis and presentation of safety data, provides guidance on the prioritisation and classification of data to be collected, as well as guidance on study design, applicable in various settings, including LMICs. The WHO Global Advisory Committee on Vaccine Safety (GACVS) provided a highly supportive assessment of the GAIA guidelines for clinical trials and considered them to be timely and useful [19]. These guidelines may also inform safety monitoring of vaccines already recommended for pregnant women (tetanus, influenza and pertussis).

The GAIA partners developed the first set of over 21 standardized case definitions of prioritized obstetric and neonatal outcomes based on the standard Brighton Collaboration process [20]. The first 10 definitions were published in a special issue of the journal *Vaccine* in December 2016. They comprise five obstetric (hypertensive disorders of pregnancy, maternal death, non-reassuring foetal status, pathways to preterm birth and postpartum haemorrhage) and five neonatal outcomes (congenital anomalies, neonatal death, neonatal infections, preterm birth and stillbirth) [21]. These were complemented with definitions and assessment algorithms of enabling terms (e.g., gestational age).

Moreover, a searchable database of terms (glossary), concept definitions and ontology of over 3000 terms related to key events for monitoring immunization in pregnancy was developed (https://evs.nci.nih.gov/ftp1/GAIA/About.html). A map of disease codes across coding terminologies, including MedDRA and ICD, was created to enable pooling of data from various sources. An online tool for automated case classification (single case or batch classification) of events according to the standardized case definitions has also been developed [12].

An investigator workshop assessing the usefulness and applicability of GAIA guidelines and case definitions in clinical trials and observational studies in LMIC, and an international consensus conference were held at the National Institute of Health (NIH) [22].

In this special issue, the next set of 11 case definitions including five obstetric outcomes (abortion, antenatal bleeding, gestational diabetes, dysfunctional labour, foetal growth retardation) and six neonatal outcomes (low birth weight, small for gestational age, neonatal encephalopathy, respiratory distress, failure to thrive and microcephaly) are published. In the light of the important public health benefit of immunization in pregnancy in particularly LMIC and the significant challenges of conducting research in low resource settings especially with pregnant women, the paper by Kochhar et al. highlights pertinent aspects of study design, regulatory and safety monitoring considerations in these settings.

The GAIA outputs are already being increasingly utilized in the field of immunization in pregnancy and maternal and child health by key stakeholders such as clinical trialists, investigators, regulators, and industry. Useful next steps would be monitoring the implementation of GAIA outputs and a structured assessment of their current field use in addition to systematic evaluation of GAIA output performance and the impact on data quality. This could guide refinement of tools, and updates of guidelines and case definitions in cyclical revision periods. A second way to stimulate scientific progress could be by developing additional guidelines and tools requested by investigators and key stakeholders, and establishing a central resource that may provide investigators and stakeholders with the standards and tools they need.

The GAIA guidelines, definitions and tools will be applicable in immunization in pregnancy pre-and post-licensure safety and pharmacovigilance surveillance systems and may help supporting enhanced surveillance and collection of safety data that can be consolidated and compared across sites, countries, and programs worldwide. A standardized approach to safety data collection and reporting is likely to improve the acceptability and implementation of immunizations in pregnancy and subsequently help reduce illness and death among pregnant women and infants globally.
